# WHO Is Recommending against the Use of COVID-19 Convalescent Plasma in Immunocompromised Patients?

**DOI:** 10.3390/life13010134

**Published:** 2023-01-03

**Authors:** Massimo Franchini, Arturo Casadevall, Michael J. Joyner, Daniele Focosi

**Affiliations:** 1 Division of Transfusion Medicine, Carlo Poma Hospital, 46100 Mantua, Italy; 2 Department of Molecular Microbiology and Immunology, Johns Hopkins Bloomberg School of Public Health, Baltimore, MD 21287, USA; 3 Department of Anesthesiology and Perioperative Medicine, Mayo Clinic, Rochester, MN 55902, USA; 4 North-Western Tuscany Blood Bank, Pisa University Hospital, 56124 Pisa, Italy

Since December 2019, SARS-CoV-2 is ravaging the globe, currently accounting for over 660 million infected people and more than 6.6 million deaths. As convalescent plasma had been successfully used in previous viral outbreaks [1], collection anhd transfusion of COVID-19 convalescent plasma (CCP) was rapidly deployed worldwide to treat patients with Coronavirus Disease-19 (COVID-19). The results of the numerous studies assessing the clinical efficacy of CCP were inconsistent and ranged from about 50% efficacy in reducing mortality to no effect, reflecting great inter-study methodological heterogeneities and inconsistencies [2]. Despite these limitations, a significant proportion of the literature supported the clinical benefit of this antibody-based treatment when administered early (within 72 h since onset of symptoms) and with a high titer of neutralizing antibodies (nAb) [2].

Interest in CCP faded during 2021, following the marketing authorization of small molecule antivirals and anti-Spike monoclonal antibodies (mAb). Nevertheless, the advent of the Omicron variant of concern (VOC) renewed the interest in CCP, because of its immune-escape to mAb-based therapies [3]. On the contrary, CPP has preserved efficacy against Omicron sublineages [4], including the recent BQ.1.1 and XBB sublineages [5]. With most humans having some immunity to SARS-CoV-2 from vaccines and/or prior infection, the Omicron variant is still life-threatening for immunocompromised (IC) patients, who are not able to mount a sufficiently protective antibody response after vaccination or infection [6]. COVID-19 in the IC population is a difficult management problem. IC patients present two interdependent problems in the form of high viral loads and reduced immunological capacity to clear the infection. A high viral load implies a high likelihood for generation of variants capable of escaping antiviral therapy.

A recent systematic review and meta-analysis including four randomized controlled trials (RCTs) and five controlled studies conducted in IC COVID-19 patients showed a clinical benefit from CCP versus standard of care (risk ratio for mortality 0.65) [7]. For such reasons, several national and international scientific societies currently recommend CCP among possible therapies in COVID-19 patients with hematological or solid cancers or other underlying congenital or acquired causes of immunosuppression. Table 1 summarizes the recommendations from six of these societies, all being favorable to the use of CCP in this particularly frail category of patients. The analysis of this table permits us to make some considerations.

- The strength of recommendation for CCP use in IC patients should be updated and upgraded, taking into account the increasing level of evidence derived from recently published RCTs. As with other fields of COVID-19 science, the research on CCP use in IC patients is rapidly evolving, and societies should create ad hoc committees that perform living systematic literature reviews to provide updated recommendations. The time from writing to publication should be expedited to avoid publishing outdated guidelines [8].
- Other continental oncohematology or transplant societies (e.g., ASH, AST, EHA, and ESOT) and international health organizations should urgently provide clear indications on CCP use in IC patients. In particular, the World Health Organization (WHO) recommendations against using CCP [9] dating from December 7, 2021, and based on outdated information, should be urgently updated to recognize the particular value of CCP in IC patients and the lack of affordable alternatives in low- and middle-income countries [10]. It took WHO 120,000 cases to declare COVID-19 a pandemic [11], and 2 years to admit that SARS-CoV-2 is airborne [12]: clearly, delays about therapeutic guidelines are unacceptable.
- The collection of CCP should restart worldwide from vaccinated people preferentially recovered from Omicron variants, in order to transfuse variant of concern (VOC)-matched high-titer CCP [4,5,13].
- New, well-designed RCTs should be restarted in order to further evaluate CCP efficacy in IC patients, in both inpatient (such as the recently re-opened REMAP-CAP arm [14]) and outpatient settings. Such trials need to incorporate the lessons learned to date including the need for high-titer CCP units and repeated dosing.

E.g., Trottier et al. reported successful treatment of protracted COVID-19 in a patient with chronic lymphocytic leukemia after 30 days of remdesivir, 20 days of Paxlovid™ and a dose of bebtelovimab [15]. Combination therapies have clear biological plausibility, but for COVID-19 in the IC, there is a dearth of high-quality clinical efficacy data. We note inconsistencies in the way that the available clinical evidence is applied to COVID-19 care. For example, none of the small molecule antiviral drugs has been tested in IC patients by RCT [16]. Molnupiravir is prescribed to vaccinated outpatients, despite no evidence that it reduces hospitalizations [17]. Similarly, mAb therapies were enthusiastically adopted for IC patients without RCT evidence or formal subgroup analysis suggesting efficacy in this population. Evusheld™ provides a good example, gaining FDA authorization for IC patients despite the PROVENT RCT authors concluding *“efficacy in these groups could not be estimated”* [18]. mAb therapies are often used regardless of serostatus, which negates the logic of replacement therapy in those who are seropositive. Remarkably, mAb use has continued despite the evidence of in vitro inefficacy across Europe [19], where EMA never withdrew a single authorization [20], while in the USA all of them were promptly withdrawn as they lost reactivity with Omicron variants [21]. It is paradoxical that many physicians do not trust in vitro surrogate markers when selecting therapy [22,23], but use them for authorizing mAbs without data for clinical efficacy.

The enthusiastic adoption of small molecule antivirals and mAbs in IC patients without hard efficacy data contrasts with the lukewarm interest in CCP as a clear example of double standards. There is considerably more evidence for clinical efficacy supporting CCP in IC patients from observational studies, formal subgroup RCT analysis and at least one RCT [7] than for any other antiviral. In contrast to mAbs, CCP is a polyclonal preparation with antibodies recognizing multiple viral epitopes and isotypes, increasing the likelihood of activity against antigenically different variants that inevitably reside in the high viral loads of IC individuals. Combining CCP with antiviral drugs makes biological sense since IC patients often have antibody deficits. Given the challenge of COVID-19 in the IC, there is a pressing need for prospective studies to evaluate combination therapies in this population. While there can be discomfort at recognizing the failure of drugs that were previously advertised as magic bullets (such as mAbs) as well as at recognizing the efficacy of treatments that were previously labeled as ineffective (such as CCP), we urge our colleagues to review the available CCP efficacy data and incorporate its use in the treatment of this vulnerable population.

**Table 1:** Summary of the guidelines on the CCP use in COVID-19 immunocompromised patients.

Guideline	Issuance	Indication	Strength of Recommendation	Certainty of Evidence	Reference
AABB	09/2022	Hospitalized: suggested use with standard care.	weak	low	[24]
		Outpatients (immunocompromised or not): suggested use with standard care.	weak	low	
NIH	12/2022	There is insufficient evidence for the panel to recommend either for or against the use of high-titer CCP for the treatment of COVID-19 in hospitalized or nonhospitalized patients who are immunocompromised. o Some Panel members would use CCP to treat an immunocompromised patient with significant symptoms attributable to COVID-19 and with signs of active SARS-CoV-2 replication and who is having an inadequate response to available therapies. In these cases, clinicians should attempt to obtain high-titer CCP from a vaccinated donor who recently recovered from COVID-19 likely caused by a SARS-CoV-2 variant similar to the variant causing the patient’s illness.	-	-	[25]
FDA	12/2021	COVID-19 convalescent plasma with high titers of anti-SARS-CoV-2 antibodies is authorized for the treatment of COVID-19 in patients with immunosuppressive disease or receiving immunosuppressive treatment, in inpatient or outpatient settings.	-	-	[26]
IDSA	3/2/2022	Recommendation 14: Among ambulatory patients with mild-to-moderate COVID-19 at high risk for progression to severe disease who have no other treatment options*, the IDSA guideline panel suggests FDA-qualified high-titer CCP within 8 days of symptom onset.	weak	low	[27]
ECIL-9	9/17/2021	Mild COVID-19: high-titer CCP is recommended in hematological patients within 72 h from symptom onset and anti-SARS-CoV-2 monoclonal antibodies not available.	weak	moderate	[28]
		Moderate COVID-19: CCP is recommended in seronegative hematological patients.	moderate	low	
NCCN (CCP obtained from subjects recovered from Omicron and previously vaccinated is preferred)	8/19/2022	Hospitalized COVID-19 cancer patients: consider high-titer CCP in immunocompromised patients, particularly those with B-cell impairment, and when anti-SARS-CoV-2 monoclonal antibodies are not available.	2A ^1^	-	[29]
		COVID-19 cancer outpatients: high-titer CCP may be beneficial in immunocompromised patients, particularly those with B-cell impairment, with persistent SARS-CoV-2 infection.	2A ^1^	-	

^1^ Category 2A: based upon lower-level evidence, there is uniform NCCN consensus that the intervention is appropriate.

## Data Availability

This manuscript generated no new dataset.

## Abbreviations

CCP: COVID-19 convalescent plasma;
nAb: neutralizing antibodies;
mAb: monoclonal antibodies;
VOC: variant of concern;
RCT: randomized clinical trial.
AABB: American Association of Blood Banks;
NIH: National Institute of Health;
FDA: Food and Drug Administration;
IDSA: Infectious Diseases Society of America;
ECIL: European Conference on Infections in Leukemia;
NCCN: National Comprehensive Cancer Network.
